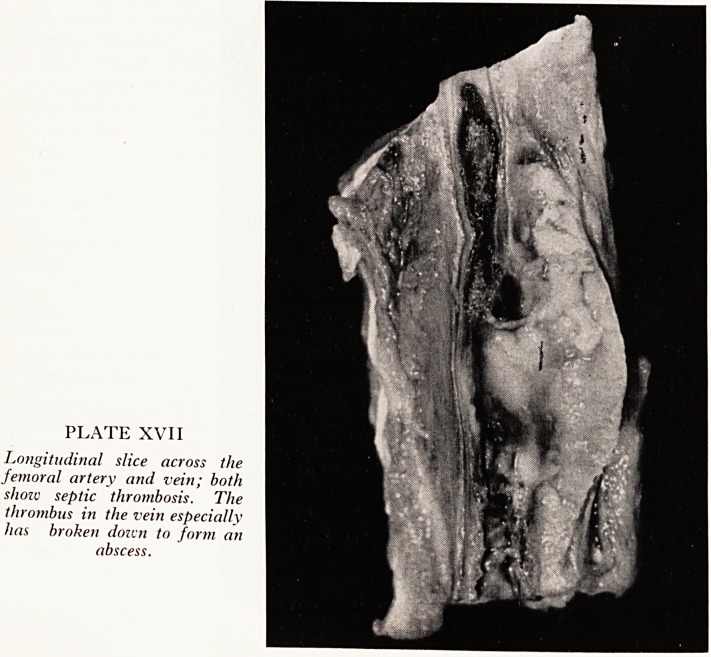# Unusual Type of Bacterial Endocarditis in a Case of Myocardial Infarction

**Published:** 1962-04

**Authors:** T. F. Hewer


### UNUSUAL TYPE OF BACTERIAL ENDOCARDITIS IN A CASE OF MYOCARDIAL INFARCTION

*A Clinico-Pathological Conference of the University of Bristol held on the 7th November, 1961*

P.M. No. 7018

CHAIRMAN: PROFESSOR T. F. HEWER

*Mr. M. W. Reece:* The patient was a retired postman, aged 65, who was admitted to hospital in February of this year, under Dr. Cates. Three weeks previously he had had a severe gripping pain in the chest going through to the back, felt very ill and went to bed. The pain eased over the next three days. A week later he had a further similar attack but this was not so bad. Following this he improved until the day of admission when he became breathless and cyanosed.

On admission his pulse rate was 104 and regular, his jugular venous pressure was raised, B.P. was 130/75 mm. Hg. He also had increased cardiac dullness, gallop rhythm, a friction rub, fine rales, a palpable liver and sacral oedema. Investigations: E.C.G. showed evidence of an anterior myocardial infarct and an X-Ray of the chest showed left cardiac enlargement. He was treated with diuretics and improved for the next eighteen days.

On the eighteenth day after his admission he got a sudden pain in his right leg below the knee, with loss of sensation in the foot, and I was asked to see him. He did not complain of the other two classical symptoms of cold or weakness. On examination, his right femoral pulse was palpable, but only high up in the groin; his foot was cold and blue with loss of power and loss of sensation. The diagnosis lay between embolus and a local thrombosis. A cardiac lesion is obvious in almost all cases of embolus and of the twenty-nine cases of embolism that we have had here in the last five years only two have been in normal rhythm and both of those had had a recent infarct. He came in this latter category. Also in favour of this diagnosis was the disappearance of the pulse at about the level of the profunda femoris, which is the site of the commonest embolus that we see.

I decided to attempt to remove the embolus, which is the practice in this hospital. A conservative approach to the treatment of emboli is becoming increasingly popular throughout the country as the results of embolectomy are, on the whole, very poor. In some centres operation is only carried out if there is impending gangrene. Our earlier figures for operative treatment are bad but we think that we are getting better results with more enthusiastic treatment, supplementing the embolectomy with intra arterial suction and retrograde flushing to remove distal clot and sometimes using regional heparinization post-operatively.

I explored his femoral artery in the groin and removed an embolus. I got a good flow back from the superficial femoral artery and the profunda. After the operation sensation and colour returned to normal in the leg but I could not feel his distal pulses. These sometimes do not return after a successful embolectomy for 2–3 days, but it probably meant that there was a distal block.

Three days later he had a further embolus, at least, we can say that he had a further similar attack and the diagnosis was either that he had thrown off a second embolus, or that he had a thrombosis at the site of arteriotomy. I re-operated on him and removed another embolus which I sent off to the pathologists who confirmed my diagnosis by saying that the clot was definitely more than three days old. On this occasion I removed clot from the superficial femoral artery and the profunda femoris and also put a sucker down to the popliteal artery to suck back propagation clot. I then opened the posterior tibial artery at the ankle and flushed out the artery with heparin saline.

As a result of this operation his foot became a lot warmer and its colour was good. Two days later his ankle pulses had returned and so this was, I think, a successful embolectomy. To prevent further thrombosis we started him on heparin immediately after operation. This may limit thrombosis but does have some disadvantages, as became apparent on the third day when he developed a haematoma in his left thigh at the site of an injection of streptomycin, and a few days later his wound started oozing and then became infected—the second hazard after heparin. This was luckily not severe and gradually subsided. I must have foreseen trouble for at this stage I departed for Scandinavia and wisely did not see the patient again.

He was then returned to the physicians but a month after his first embolism he had another one, with a similar onset of pain and coldness. He was again seen by the Surgical Unit. His femoral artery was re-opened, an embolus was seen and the procedures repeated. Of course his common femoral artery had been opened twice before and was not in as good condition as it had been. By the second post-operative day it was obvious that there was no return of circulation to the leg. It was decided that if he was not to lose his leg a further attempt should be made. His femoral artery was re-opened, clot removed, the artery flushed through and heparin inserted and a small vein patch was used to close the incision in the artery. At the end of the operation there was good pulsation in the common femoral and also the profunda.

I think I should emphasize at this point the reason for repeated and probably unsucessful embolectomies. If the profunda is blocked as well as the superficial femoral the blood supply to the thigh is extremely poor and the chances of getting a good above-knee amputation stump are small. The wound does not heal and becomes infected; the mortality rate is extremely high. If we could keep the profunda clear there was a good chance of getting a healed stump and a live patient. In this case the embolectomy did not save the leg, which became gangrenous and had to be amputated, but was successful in preserving the profunda which bled profusely at the time of amputation.

We were a little worried at this time because the groin wound was infected with a Proteus which was sensitive to chloramphenicol and a course of this was started. He improved for a while but developed a pustular rash in the groin, of a type that was going round the ward at this time. From it a Pyocyaneus, sensitive to chloramphenicol, terramycin and neomycin was obtained. On the ninth day after his last embolectomy and four days after his amputation he was found sitting in a pool of blood. He was obviously shocked and had had a secondary haemorrhage from his infected groin wound. This was re-explored and his femoral artery ligated. Despite a groin wound infection from which Strep. faecalis, Proteus and Coliforms were isolated he slowly improved for three weeks until he started getting rigors. During the first rigor his temperature rose to 103°F; he looked ill, cold and clammy, with a poor pulse and a B.P. of 100/60. Three days later he had a further rigor and a blood culture grew a Proteus sensitive to chloramphenicol, furadantin and kanamycin. He was started on a course of the last. Four and five days later he had further rigors and from a blood culture during each of these Proteus was again grown.

When we looked round for a source of this Proteus we found that the amputation stump was tender; its circulation was not very good and we assumed that the infected emboli were coming from the femoral vein stump. As intravenous chloramphenicol, penicillin and probenecid did not prevent further rigors his external iliac vein was ligated. Again he improved and was returned to the care of the physicians. However, his condition deteriorated the following day and a day later he died.

*Prof. Hewer:* There is a note on the 8th May—"no clinical evidence of bacterial endocarditis". I think that was perhaps made in the surgical ward, was it? Did they have any evidence in the medical ward?

*Dr. R. S. Crow:* Unfortunately I do not remember this patient in the early stage of his illness. As a matter of fact I was probably away for a large part of the time. When he came in he had had a myocardial infarction and was suffering from congestive heart failure and I am surprised that he was not treated with anticoagulants, as a prophylactic against thromb-embolic complications. Whatever one thinks about the routine treatment of myocardial infarction with anticoagulants, and there are arguments on both sides, it is much more certain that in a patient who has had an infarction and who develops heart failure, the risk of thrombo-embolic complications is high and therefore anticoagulants should be used. The usual reason for deciding against anticoagulants is that there are contra-indications such as peptic ulceration, or possibly even a pericardial effusion. I cannot tell from the notes why he did not have them. Indeed my first recollection of the patient is that on 16th May as a result of rigors and pyrexia persisting for a considerable time I was asked to see him then in the surgical ward; someone, the surgeon I suppose, had noted no evidence of bacterial endocarditis—spleen not enlarged, fundi normal, no splinter haemorrhages—and at the same time had noted: "right fingers always clubbed". So I would not press the finger clubbing. The patient was jolly ill at that time, I remember, and was not really very clear in the head; it was difficult to get any details from him when I asked him whether his hands had always been like that. I assumed it was a recent development, caused by bacterial endocarditis. There was no other evidence of that, so that we cannot pin anything down. However, the man was obviously extremely ill; it looked as though he had septicaemic infection which multiple antibiotics were not holding. I did not really think it would retrieve the situation to transfer him back to the medical ward, and obviously we did not do him any good; antibiotics were continued, but he only survived two more days. Certainly the diagnosis of endocarditis had been considered by a house surgeon and there was this clubbing. No clubbing was noted by a very competent house physician on admission and therefore I think it was not there. However, the house surgeon's note seems to indicate that his right hand fingers had always been clubbed, which is a possibility but I am thinking he did not in fact have clubbing of the fingers at the start.

*Dr. A. B. Raper:* Could I check on that question of clubbing, please? I wonder if the physicians would agree with me that you can watch clubbing appear from no clubbing, and you can ask the patient when the clubbing appears "Have your fingers always been like this?", and he will say "Yes" and swear that they always have.

*Dr. Crow:* Yes, I am sure that is true. They usually have not noticed any difference and if they have they think that is what their hands have always looked like.

*Dr. J. M. Naish:* They think it is a slight on their powers of observation if you suggest there is any change.

*Professor Hewer:* Any questions before we come to the post-mortem? It is quite clear that there is a focus of infection here somewhere.

*Dr. N. G. Sanerkin:* At necropsy I was not really convinced that there was any clubbing, but personally I feel it is extremely difficult to detect early finger-clubbing after death. He was a well-developed elderly man with considerable recent wasting. His right lower limb had been amputated above the knee and the amputation wound was breaking down with considerable gangrene around it. There was an operation wound across the right groin along the ilio-femoral vessels; centrally this wound had broken down and was infected, being covered by thin purulent exudate with a foul smell. A healing operation wound was also present in the right iliac fossa. The abdominal cavity showed fibrinous and recent fibro-vascular adhesions around the appendix, secondary to infection of the tissues around the iliac vessels. The pericardial cavity had been obliterated by an organizing fibrinous exudate.

PLATE XIV

*Left ventricle opened up to show old antero-apical infarct and mural thrombus. Vegetations are present on two of the aortic valve cusps. The recent infarct is obscured by the reflected ventricular flap.*

PLATE XV

*Left ventricular wall above and behind the posterior papillary muscle. The depressed area along the inner third of the myocardium represents a recent organizing infarct.*

PLATE XVI

*Atrial septum viewed from the right side; tricuspid valve below. 1.—Patent foramen ovale, normally kept closed by higher pressure in the left atrium. 2.—Defect in septum secundum. 3.—Septum primum; defects at this site often involve the atrioventricular valves.*

PLATE XVII

*Longitudinal slice across the femoral artery and vein; both show septic thrombosis. The thrombus in the vein especially has broken down to form an abscess.*

The left ventricle (Plate XIV) was dilated and slightly hypertrophied and showed an old antero-apicalmyocardial infarct, where the ventricular wall was thinned and extensively replaced by coarse fibrous tissue. This infarct almost certainly dates from January when he had his first episode of cardiac pain, which makes it about four months old. It was due to occlusive thrombosis of the anterior descending coronary artery which was severely atheromatous and stenotic to begin with. A more recent and smaller infarct (Plate XV) was found posteriorly in the left ventricle, probably about three weeks old; fibrosis had not yet occurred in this infarct, and it appeared gelatinous and depressed due to replacement of the necrotic muscle by granulation tissue. To account for it there was severe stenosis of the right coronary artery at a point about 6 cm. from its origin due to haemorrhage into a patch of atheroma. The circumflex artery, which may provide collateral supply to the myocardium posteriorly, was severely atheromatous and stenosed as well. The endocardium over the old infarct was covered by thick mural thrombus (Plate XIV) with a cystic cavity in its lower part which was lined by purulent exudate and at its attachment to the endocardium contained an adherent ante-mortem blood-clot presumably following haemorrhage from the infected endocardium. A section from this area showed the coarse myocardial fibrosis of a healed infarct and the cavity in the mural thrombus which was lined by a pyogenic membrane in which occasional Gram negative bacilli were noted, indicating that bacterial infection of the endocardium had occurred in relation to the mural thrombus. Proteus was grown from a swab taken from this area.

It was interesting to find firm vegetations on the aortic valve (Plate XIV). To the naked eye these vegetations did not appear at all infected but seemed to be ordinary terminal or "marantic" thromboses. On closer inspection both vegetations showed a papillary or fern-like structure and histological examination showed a simple mural thrombosis, without any evidence of infection, occurring on the natural filiform excrescences normally found on this valve, that are called Lambl's excrescences. These may sometimes grow and enlarge through platelet and fibrin deposition to produce "papillary tumours" of the aortic valve. Exactly such a phenomenon had been going on here.

Incidentally, this patient had a patent foramen ovale and a septum secundum type of atrial septal defect as well (Plate XVI). Patency of the foramen ovale is an extremely common finding at necropsy and is obviously of no clinical significance whatsoever unless the patient has severe pulmonary hypertension, in which case a right-to-left shunt may develop through the otherwise closed valvular patency. Atrial septal defects are of considerable clinical importance, especially now that they can be surgically repaired. The septum secundum defect noted here was quite small and does not appear to have produced any significant left-to-right shunt, since the right heart chambers were neither dilated nor hypertrophied. The site where septum primum defects occur is shown in the same figure; these are much more deforming than septum secundum defects since they often involve the atrioventricular rings and valves.

The aorta was severely atheromatous and showed several mural thrombi in its abdominal portion. The lowest of these had extended into the right common iliac artery and had completely occluded its orifice but it is evident that this happened more recently than the episodes of embolism, and red propagation clot was found between the occluded orifice and the older organizing thrombus further down.

Following the repeated right femoral arteriotomies for embolectomy the operation wound became infected with Proteus and Pyocyaneus. The ilio-femoral vessels (Plate XVII) were found to be thrombosed and infected; in many stretches liquefied pus could be seen within the thrombi which were breaking down, and post-mortem culture from these grew Proteus. The purulent thrombophlebitis understandably led to a Proteus septicaemia during the course of which the ventricular mural thrombus itself became infected, opening the way for further mischief via septic emboli.

It is obviously difficult to be sure if all the episodes of femoral occlusion were embolic and not merely the result of locally-formed thrombi. I re-examined the sections of the embolectomy thrombi and found that they were mostly in fragments, and unless one gets the entire thrombus intact it can be very difficult to determine whether it was embolic or locally-formed. I should imagine it might be much easier for the surgeon to decide during the operation from the appearance of the occluding thrombus, since an embolus would look different as he took it out.

*Mr. Reece:* In this case it was a normal-looking vessel and old clot was removed.

*Prof. Hewer:* You would probably be able to say whether the thrombus was formed locally by observing direction of the layers of thrombus.

*Dr. Sanerkin:* We can therefore assume that this patient did have repeated emboli to the femoral artery. Embolism had also occurred to the spleen and kidney, causing infarcts, as well as terminally into the superior mesenteric artery. Some of these infarcts had occurred before and some after the infection of the ventricular mural thrombus. The splenic and many of the renal infarcts were fairly old and showed advanced degrees of resorption and organization, without evidence of any infection; they had developed before the onset of septicaemia. Many of the recent renal infarcts were, however, septic and had obviously resulted from lodgment of infected emboli in the renal arteries. The superior mesenteric embolism was a pre-terminal event, for the intestine showed no change apart from small recent mucosal haemorrhages confined to the tips of mucosal folds.

This, then, is the story of a man with cardiac infarction developing thrombo-embolic complications, further complicated by secondary infection of his ventricular mural thrombus, a most unusual phenomenon.

*Professor Hewer:* It amounts to bacterial endocarditis on top of mural thrombus instead of on the valve cusp, blood-borne from the infected vein in the leg.

*Dr. O. C. Lloyd:* Was the Proteus the only organism you grew from the mural thrombus in the heart, and did you grow Proteus also from the ones in the aorta?

*Dr. Sanerkin:* We grew Proteus and a few Coliforms from the septic vessels in the leg.

*Dr. Lloyd:* Yes, and the heart?

*Dr. Sanerkin:* The same, Proteus.

*Dr. Lloyd:* and Pyocyaneus?

*Dr. Sanerkin:* No Pyocyaneus.

*Dr. Crow:* It is interesting if these all were emboli that they should all land at the same place. Is this because of the flow, or what? Can you tell me?

*Mr. Reece:* The common femoral embolus is the commonest peripheral one that we see. In our series over half are at this site. The larger ones are less common and the more peripheral ones often produce few signs and may be missed.

*Dr. Crow:* How many emboli do you see on the left?

*Mr. Reece:* I have not checked whether a right femoral embolus is any commoner than a left. Certainly the fourth episode was due to thrombosis and I suppose the third could also have been. It may be difficult at operation to tell between an embolus with a lot of propagated clot and a thrombosis.

*Professor Hewer:* It is arguable, is it not, that after the first one the others were local thrombosis?

*Mr. Reece:* I took the second clot to the pathologists and they said it was more than three days old, that is before the first operation.

*Dr. Crow:* I think that anyone with myocardial infarction and heart failure should have anticoagulants whatever one thinks about the routine treatment for infarction.

*Mr. Reece:* We always give patients anticoagulants if we treat them conservatively. There is little evidence that it diminishes the incidence of gangrene but it probably lessens the risk of further emboli.

## Figures and Tables

**Figure f1:**
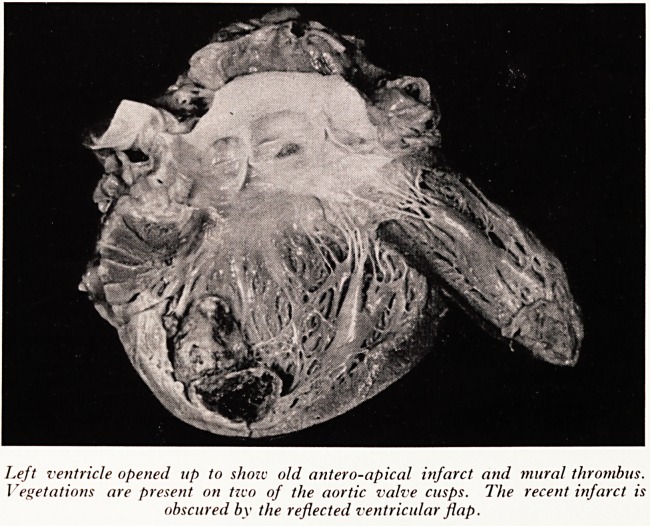


**Plate XV f2:**
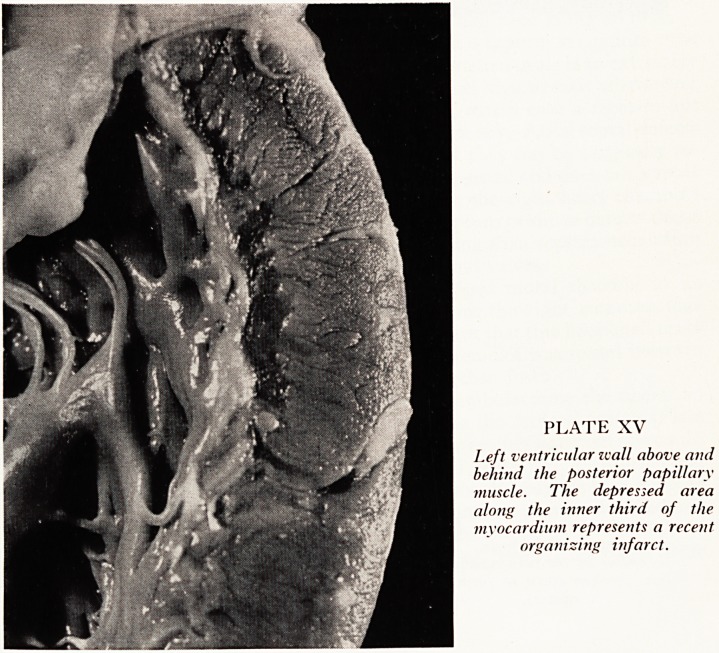


**Plate XVI f3:**
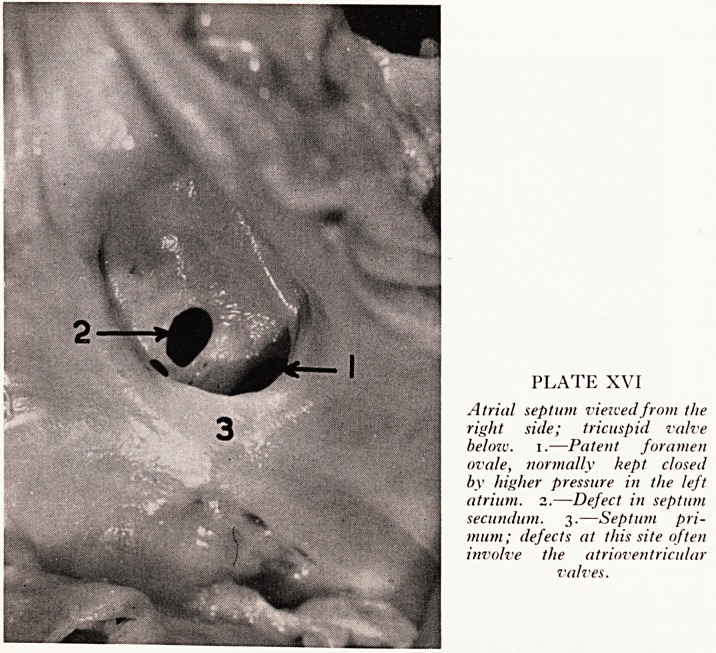


**Plate XVII f4:**